# Oral Bioavailability of Kinsenoside in Beagle Dogs Measured by LC-MS/MS: Improvement of Ex Vivo Stability of a Lactone-Containing Compound

**DOI:** 10.3390/pharmaceutics10030087

**Published:** 2018-07-09

**Authors:** Xin Zhang, Ming Jin, Yuping Liu, Qimingxing Chen, Luqin Si, Gao Li, Yonghui Zhang, Jiangeng Huang

**Affiliations:** School of Pharmacy, Tongji Medical College, Huazhong University of Science and Technology, Wuhan 430030, China; siriusstar.zx@gmail.com (X.Z.); DuesKing24680@gmail.com (M.J.); 18211030063@fudan.edu.cn (Y.L.); chenqmx.11@gmail.com (Q.C.); luqin_si@hust.edu.cn (L.S.); gao_li@hust.edu.cn (G.L.); zhangyh@mails.tjmu.edu.cn (Y.Z.)

**Keywords:** kinsenoside, LC-MS/MS, beagle dog, bioavailability

## Abstract

Kinsenoside (KD), an active compound isolated from *Anoectochilus roxburghii*, has demonstrated multiple pharmacological activities including hepatoprotection, antihyperliposis, antihyperglycemia, and antiosteoporosis. To the best of our knowledge, there are no available data concerning its preclinical pharmacokinetics and bioavailability in beagle dogs. To support preclinical pharmacokinetic and bioavailability study, a reliable LC-MS/MS method was developed for KD concentration measurements in beagle dog plasma. The chromatographic separation was achieved on a Waters Atlantis^®^ Hilic Silica column with an optimum mobile phase consisting of 5 mM ammonium acetate in water (pH 3.0 adjusted with acetic acid) and acetonitrile at a flow rate of 0.2 mL/min. Mass spectrometric analyses were carried out by monitoring multiple reaction monitoring transitions at *m*/*z* 265.2→102.9 for KD and *m*/*z* 174.0→128.0 for l-phenyl-d_5_-alanine-2,3,3-d_3_ (IS). The stability of KD in beagle dog whole blood and plasma was systematically evaluated. Lowering the temperature played a more critical role in stabilizing KD than decreasing the pH and adding esterase inhibitors, indicating that the major reason for instability of KD was probably due to chemical hydrolysis rather than esterase-mediated degradation. The currently developed method was validated and applied to a pharmacokinetic and bioavailability study of KD in beagle dogs following oral administration at a dose of 3 mg/kg. The absolute oral bioavailability for KD was determined to be 27.6%. Compared with typical glycosides, KD has a better bioavailability and is suitable for developing an oral dosage form.

## 1. Introduction

Kinsenoside (KD) is a major known active compound isolated from *Anoectochilus roxburghii* (Orchidaceae), a traditional herb used in many Asian countries for medicinal and culinary purposes [1]. Extensive preclinical research in KD was carried out due to its multiple pharmacologic activities. Previous investigations showed that KD possessed hepatoprotective properties by decreasing the levels of the cytosolic enzymes such as lactate dehydrogenase (LDH), glutamic-oxalacetic transaminease (GOT), and glutamate pyruvic transaminease (GPT) [2,3]. KD was also reported to exert antidiabetic, antioxidant, and antiosteoporosis activities [4,5,6]. In addition, KD demonstrated vascular protective properties due to oxidative stress inhibition and vascular endothelial structure maintenance [7]. More recently, our colleagues reported that KD exhibited immunosuppression against autoimmune hepatitis by disrupting dendritic-cell-induced cross-priming of CD8^+^T cell response [8].

Although KD presents promising therapeutic potential, its pharmacokinetic properties remain largely unexplored. Pharmacokinetic and oral bioavailability studies provide valuable information on drug candidates to define a proper dosage form during the drug development stage. To the best of our knowledge, there are no available data concerning its preclinical pharmacokinetics and bioavailability in beagle dogs. To support preclinical pharmacokinetic study, a reliable bioanalytical method was required. Most of the previous analytical methods reported for the quantitation of KD utilized high-performance liquid chromatography with ultraviolet or evaporating light scattering detectors [3,4,8,9]. Due to their poor sensitivity, such methods are not suitable for in vivo pharmacokinetic research. In 2015, Shaheed Ur Rehman et al. developed a liquid chromatography–tandem mass spectrometry (LC-MS/MS) method employing a Waters Acquity UPLC BEH C_18_ column for metabolic stability investigation of KD [10]. However, reverse-phase columns demonstrated obvious shortcomings for highly polar compounds such as KD due to their poor retention. Subsequently, a hydrophilic interaction liquid chromatography–tandem mass spectrometry (HILIC-MS/MS) method was developed for the determination of KD in rat plasma [11]. In this paper, the authors paid attention to the plasma stability issue of KD and specific strategies were employed to improve the stability of the analyte of interest. However, the stability of KD in rat whole blood has not been investigated. Although stability in plasma is considered as an indicator of the analyte stability in blood, there are exceptions for which the stability behavior of the drug in blood is different from that in plasma [12]. Moreover, the stability of KD in whole blood and plasma from different species such as beagle dog remains unknown. Therefore, the current work performed systemic stability assessment of KD in beagle dog whole blood and plasma and thereafter established blood sampling and treatment procedures to ensure a reliable concentration determination of the analyte. Finally, the developed and validated LC-MS/MS method was applied for pharmacokinetic and bioavailability study of KD in beagle dogs for the first time.

## 2. Materials and Methods

### 2.1. Chemicals and Reagents

Kinsenoside was isolated from *Anoectochilus roxburghii* and purified in our own lab. The purity of kinsenoside was >98%, as measured by a high-performance liquid chromatography (HPLC) system with an evaporative light scattering detector. l-phenyl-d_5_-alanine-2,3,3-d_3_ (internal standard, IS) was obtained from CDN Isotopes (Pointe-Claire, QC, Canada). 2,2-Dichlorovinyl dimethyl phosphate (DDVP) and phenylmethanesulfonyl fluoride (PMSF) were purchased from Sigma-Aldrich (St. Louis, MO, USA). Sodium fluoride (NaF) was obtained from Sinopharm Chemical Reagent Co. Ltd. (Shanghai, China). HPLC-grade acetonitrile (ACN) was purchased from Fisher Scientific (Fair Lawn, NJ, USA). K_2_EDTA-containing vacutainer tubes (3 mL) and 22 G IV catheters (0.9 mm × 25 mm) were obtained from BD Biosciences (Franklin Lakes, NJ, USA). All other reagents were of analytical grade.

### 2.2. LC-MS/MS Conditions

Analysis was performed using a Shimadzu Prominence UFLC system (Shimadzu Corporation, Kyoto, Japan) coupled with an API 4000 QTrap^®^ triple quadrupole mass spectrometer (AB Sciex, Foster City, CA, USA) equipped with an electrospray ionization (ESI) source.

Chromatographic separation was achieved on a Waters Atlantis^®^ Hilic Silica column (2.1 mm × 100 mm, 3 μm) preceded by a 0.22 μm in-line filter (Waters Co., Milford, MA, USA). The mobile phase consisted of 5 mM ammonium acetate in water (pH 3.0 adjusted with acetic acid) (Solvent A) and ACN (Solvent B). A gradient profile was applied by starting at 90% B, decreasing to 50% B within 2 min, holding 50% B for 3 min, and returning to initial 90% B within 0.1 min, followed by an equilibration of 1.9 min, resulting in a total run time of 7 min. The column temperature was maintained at 30 °C and the injection volume was 10 μL.

Mass spectrometric detection was carried out under the positive ion electrospray mode using multiple reaction monitoring (MRM), monitoring the precursor-to-product ion transitions of *m*/*z* 265.2→102.9 and *m*/*z* 174.0→128.0 for KD and l-phenyl-d_5_-alanine-2,3,3-d_3_ (IS), respectively. The source parameters were optimized as follows: curtain gas, 30 psi; collision gas (CAD), medium; ionSpray voltage, 5500 V; temperature, 600 °C; nebulizer gas, 30 psi; auxiliary gas, 30 psi; and interface heater, on. The optimum values for compound-dependent mass spectrometric parameters including declustering potential (DP), collision energy (CE), entrance potential (EP), and cell exit potential (CXP) were set at 36, 15, 10, and 10 V, respectively, for KD and at 52, 24, 10, and 15 V, respectively, for IS. Data acquisition and processing were controlled by Analyst 1.6.1 software (AB Sciex, Foster City, CA, USA).

### 2.3. Preparation of Calibration Standards, Quality Control (QC) Samples, and Esterase Inhibitor Solutions

The stock solutions of KD and IS were prepared in ACN–water (1:1, *v*/*v*) at concentrations of 2.5 mg/mL and 1 mg/mL, respectively. Working solutions containing 125, 250, 500, 1250, 2500, 12,500, 25,000, and 50,000 ng/mL KD or 500 ng/mL IS were prepared by serial dilution with ACN–water (1:1, *v*/*v*) from corresponding stock solutions. Both stock and working solutions were stored at −80 °C until use.

To prepare calibration standards, 5 μL working solutions were added to 120 μL aliquots of blank dog plasma, yielding a series of calibration standards with final concentrations of 5, 10, 20, 50, 100, 500, 1000, and 2000 ng/mL for KD. QC samples were prepared in a similar manner at three different levels (15, 150, and 1500 ng/mL). Esterase inhibitor solutions containing 500 mM DDVP, NaF, and PMSF were prepared in ACN–water (1:1, *v*/*v*), water, and dimethylsulfoxide (DMSO), separately. All three esterase inhibitor solutions were stored at −80 °C until use.

### 2.4. Sample Preparation

After thawing at 4 °C, 50 μL aliquots of calibration standards, QC samples, and actual plasma samples were spiked with 25 μL IS working solution. Thereafter, 250 μL acetonitrile containing 0.1% acetic acid (AA) was added. The mixture was vortexed for 5 min and then centrifuged at 15,000× *g* for 10 min at 4 °C. Subsequently, 200 μL of the supernatant was aspirated and evaporated to dryness under nitrogen flow at 4 °C. The dry extracts were reconstituted in 100 μL ACN–water (9:1, *v*/*v*) and used for LC-MS/MS analysis.

### 2.5. Stability Evaluation in Dog Whole Blood and Plasma

The stability of KD in fresh dog whole blood and plasma was studied by decreasing pH, lowering temperature, and adding different esterase inhibitors. All experiments were performed in triplicate using three different lots of fresh dog whole blood or plasma. To decrease the pH of the matrixes, 1% formic acid (FA) or 1% AA was individually added to the fresh blank whole blood or plasma with a ratio of 1:9. Likewise, DDVP, NaF, and PMSF were separately added to fresh blank whole blood or plasma to obtain a final concentration of 50 mM. After 5 min pre-incubation, KD was spiked with pretreated whole blood or plasma to obtain final concentrations of 15, 150, and 1500 ng/mL. The whole blood mixture was maintained at room temperature or on wet ice for 2 h except for samples of time zero, which were centrifuged immediately to collect plasma, while the plasma mixture was maintained at room temperature or on wet ice for 0, 2, and 6 h. Then, the obtained plasma samples were cleaned up using the previously mentioned sample preparation procedures. The remaining percentage of KD was calculated by dividing the plasma concentration of KD under different incubation conditions by the concentration of time zero samples [13].

### 2.6. Method Validation

The method validation was conducted according to FDA’s Guidance for Bioanalytical Method Validation. Specificity, the limit of detection (LOD), lower limit of quantification (LLOQ), calibration curve, accuracy, precision, recovery, matrix effect, carry-over, stability, and dilution integrity were all investigated [14].

The specificity was assessed by comparing the blank dog plasma chromatograms achieved from six different sources with that of plasma spiked with KD at the LLOQ level to exclude interfering peaks at the retention times of KD and IS. LOD was defined as the concentration that produced a signal three times above the noise level. LLOQ was defined as the lowest concentration with acceptable accuracy and precision with a signal-to-noise ratio above 5. The calibration curves were assayed and constructed in three different batches by plotting the peak area ratios of the analyte to IS against the nominal concentrations using a 1/*x*^2^ weighting scheme. The deviations of each backcalculated concentration should be within 85–115% of the nominal values except for LLOQ, for which 80–120% was permitted. Intra-day and inter-day accuracy and precision were assessed by analyzing six replicates of LLOQ and QC samples on three different runs. The extraction recovery was evaluated from the ratio of the mean peak areas of six replicates of extracted QC samples compared with those of post spiked blank plasma samples with the equivalent concentration of analyte and IS. The matrix effect was determined by calculating the ratio of the mean analyte peak areas of the post spiked QC samples to the response from the same concentration in ACN–water (1:1, *v*/*v*). Carry-over was investigated by analyzing one blank plasma sample after the highest concentration of calibration standard.

The stability of stock solutions (2.5 mg/mL for KD and 1 mg/mL for IS) and working solutions (125 and 50,000 ng/mL for KD and 500 ng/mL for IS) was evaluated at room temperature for 6 h and at −80 °C for 31 days. The matrix stability was evaluated from three replicates of each QC sample stored in different conditions using freshly prepared calibration standards. The QC samples were analyzed after storage on wet ice for 2 h (short-term stability) and at −80 °C for 21 days (long-term stability). The freeze–thaw stability was determined after freezing at −80 °C and thawing on wet ice for three cycles. To evaluate the post-preparative stability, QC samples were analyzed after being held in the autosampler for 24 h at 4 °C. The samples for stability evaluation were considered stable if the deviation was within ±15% of the nominal concentration.

The dilution integrity was evaluated by diluting plasma samples spiked at 16,000 ng/mL with blank dog plasma with a ratio of 1:9 to a final concentration of 1600 ng/mL. The dilution integrity was considered acceptable if the precision was within 15% and the accuracy was within 85–115%.

### 2.7. Pharmacokinetic and Bioavailability Study of KD in Beagle Dogs

The validated method was applied to a preclinical pharmacokinetic and bioavailability study of KD in beagle dogs following intravenous and oral administration. The study protocol (Project ID: HUST-D-201802004) was approved on 5 March 2018 by the Animal Ethics Committee in Tongji Medical College, Huazhong University of Science and Technology (Wuhan, China). Six beagle dogs (3 males and 3 females) weighing 10.0 ± 2.0 kg were purchased from Ruikesen Experimental Animal Co., Ltd. (Wuhan, China). All dogs were individually housed at a temperature of 25 °C and 50% relative humidity under a 12 h light/dark cycle. A laboratory standard diet and water were provided *ad libitum*. After overnight fasting with unlimited access to water, the six beagle dogs were randomly divided into two groups. One group was intravenously injected via lateral saphenous vein with 3 mg/kg KD, while the other group was given a single oral dose of 3 mg/kg KD. After a 7-day washout period, each group received the other treatment. For intravenous administration, blood samples (approximately 1 mL) were collected via cephalic vein into 3 mL K_2_EDTA-containing vacutainer tubes at pre-dose and at 0.083, 0.25, 0.5, 0.75, 1, 1.25, 1.5, 2, 2.5, 3, 4, and 5 h after administration. For oral administration, blood samples (approximately 1 mL) were collected at pre-dose and at 0.25, 0.5, 0.75, 1, 1.25, 1.5, 2, 2.5, 3, 4, 5, 6, and 7 h post-dose. Blood samples were maintained on wet ice and centrifuged under 4 °C at 3500 × *g* for 5 min to obtain plasma. Plasma samples were immediately transferred to pre-cooled plastic Eppendorf tubes and stored at −80 °C until analyzed.

### 2.8. Pharmacokinetic Parameter Calculation

The pharmacokinetic parameters were calculated using noncompartmental analysis by Phoenix WinNonlin software (Version 6.4, Pharsight Corporation, Princeton, NC, USA). The maximum plasma concentration (C_max_) and time to reach C_max_ (T_max_) were obtained directly from the plasma concentration time course. The area under the plasma concentration versus time curve (AUC) from time zero to the last measured time point (AUC_0–t_) and from time zero to infinity (AUC_0–∞_) were estimated using the log-linear trapezoidal rule, while the elimination half-life (t_1/2_), the total body clearance (CL), and the apparent volume of distribution (V_d_) were processed by Phoenix WinNonlin software. Oral bioavailability (F) was calculated using the formula F = (AUC_po_/AUC_iv_) × (Dose_iv_/Dose_po_).

### 2.9. Statistical Analyses

Statistical analyses were carried out using GraphPad Prism 6.0 (GraphPad Software Inc., USA). Differences between the two groups were analyzed using Student’s *t*-test. A value *p* <0.05 was considered statistically significant.

## 3. Results and Discussion

### 3.1. Method Optimization

**Figure 1:**
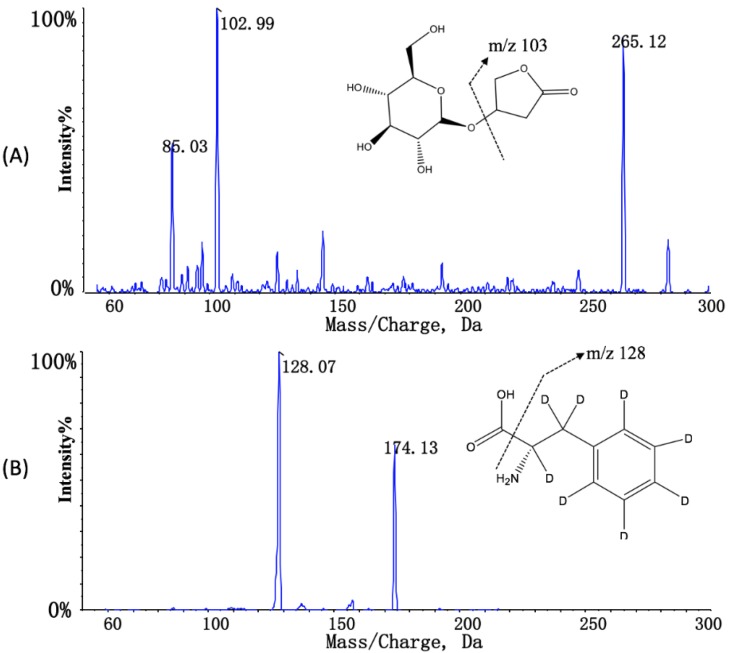
The chemical structures and characteristic diagnostic product ions of (**A**) kinsenoside and (**B**) internal standard.

The mass spectrometric (MS) parameters were optimized to maximize the MS response. By direct infusion of 100 ng/mL KD or IS neat solution prepared in ACN–water (1:1, *v*/*v*), MS spectra revealed peaks for the most abundant protonated molecular ions [M + H]^+^ *m*/*z* 265.2 and 174.0 under positive mode for KD and IS, respectively. In the product scan mass spectrum, the major fragment ions observed in each product spectrum were *m*/*z* 102.9 and 128.0 for KD and IS, respectively. The chemical structure and characteristic diagnostic product ions of KD and IS are presented in Figure 1.

The chromatographic conditions including column type, mobile phase composition, and gradient profile were optimized for improved analyte retention and chromatographic peak shape on the basis of the previously published method [11]. As expected, poor retention on reversed-phase C18 and C8 columns is one of the most challenging issues to determine highly polar compounds such as KD. Therefore, an HILIC column was used because of its better retention for KD. Different from the previous method, at least 5% polar solvent was maintained in the whole gradient condition to ensure that silica particles were hydrated. Moreover, 5 mM ammonium acetate in water adjusted to pH 3.0 using acetic acid instead of 0.1% formic acid in distilled water was employed as the aqueous phase to improve the analytical reproducibility and peak shape of KD during LC-MS/MS analysis.

The previously published method employed an acidified mixture of acetonitrile and methanol as the precipitating solvent. However, the resultant supernatant directly obtained after precipitation was found to be inappropriate due to the bad peak shape of the analyte (data not shown). Since chromatographic behavior on HILIC columns was readily affected by the sample solvent, sample clean-up was further optimized in the current assay. Compared with the previously reported sample treatment method, a procedure including evaporation to dryness under nitrogen flow at 4 °C followed by reconstitution in ACN–water (9:1, *v*/*v*) was employed to achieve a better peak shape for KD.

As for the choice of the internal standard, a stable-isotope-labeled IS of the analyte is preferred for LC-MS/MS analysis. However, such IS of KD is not commercially available. Several compounds with high polarity such as isoorientin and galuteolin were tried first. The retention time of these two compounds was close to that of KD, but significant ion suppression was observed for isoorientin and galuteolin (data not shown). Nicotine, a liquid alkaloid used as an internal standard in the previously reported analytical method, was also found to be unsuitable under our evaporation procedure due to its high vapor pressure. It may cause cross-contamination, losses during the evaporation processes, and ubiquitous presence at low but measurable levels in biological samples [15]. The function group (alicyclic amine) of nicotine contains a lone pair of electrons on the nitrogen atom and may suffer stability issues in biological samples [16]. Besides this, the retention behavior of nicotine is not similar to that of KD under the current chromatographic condition. Since amino acid possesses high polarity and it could be retained on the HILIC column, l-phenyl-d_5_-alanine-2,3,3-d_3_, a deuterium-labeled amino acid, was tested as the IS. The ionization efficiency of KD and l-phenyl-d_5_-alanine-2,3,3-d_3_ was different, but the main purpose of utilizing ISs in LC-MS/MS bioanalysis was to improve the accuracy and precision of quantitation as well as the robustness of bioanalytical methods. In the current method, both the matrix factor and the IS-normalized matrix factor of KD were close to 1.0, which indicated that there were no co-eluting matrix components that impacted the ionization efficiency of KD. Furthermore, the recovery of KD was consistent with that of IS. Finally, l-phenyl-d_5_-alanine-2,3,3-d_3_, a deuterium-labeled amino acid, was selected as IS due to the similar retention behavior and no appreciable matrix effect.

### 3.2. Stability Evaluation of KD in Dog Whole Blood and Plasma

**Figure 2:**
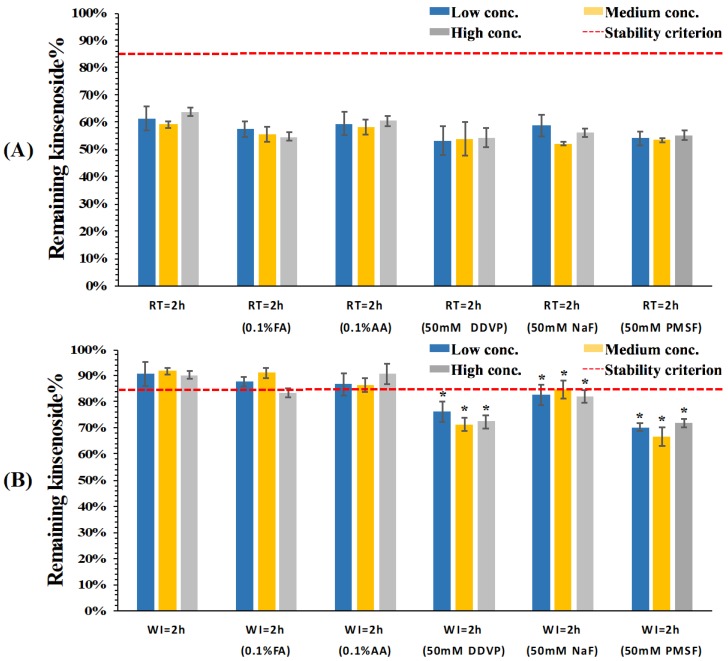
Remaining percentage of kinsenoside in fresh beagle dog whole blood after 2 h incubation under (**A**) room temperature and (**B**) wet ice conditions (*n* = 3). The red dash lines indicate the stability criterion (85% of the nominal analyte concentrations). FA: formic acid; AA: acetic acid; DDVP: 2,2-Dichlorovinyl dimethyl phosphate; NaF: sodium fluoride; PMSF: phenylmethanesulfonyl fluoride. * *p* < 0.05 compared with whole blood samples incubated on wet ice after 2 h.

KD has been proven to be unstable in rat plasma (ex vivo), most probably owing to a lactone group in the aglycone ring [11]. Lactone-containing compounds were proved to undergo both chemical hydrolysis and enzymatic breakdown caused by the action of esterases in matrixes [17,18]. Since the stability of KD in dog whole blood and plasma remains unknown, the stability of KD in such biological matrixes was systemically studied and several strategies including decreasing pH, lowering temperature, and adding different esterase inhibitors were tried to overcome the stability issue of KD. As expected, KD was found to be unstable in both dog whole blood and plasma. As shown in Figure 2A, only 60% KD remained in dog whole blood after 2 h incubation at room temperature. Moreover, pH controlling and addition of different esterase inhibitors had little effect on stabilizing KD in dog whole blood when the samples were incubated at room temperature. Subsequently, whole blood samples were incubated on wet ice using the same strategies to investigate the influence of temperature on the stability of KD in dog whole blood. Interestingly, temperature played a more crucial role than did pH control and addition of esterase inhibitors in stabilizing KD in dog whole blood. As shown in Figure 2B, around 90% KD remained after 2 h incubation on wet ice at three different concentration levels. In addition to lowering temperature, which is a common strategy used to stabilize analytes in biological matrixes [19], the addition of esterase inhibitors and adjusting pH offer effective approaches to stabilize the ester-containing analytes. Unexpectedly, the combination of different esterase inhibitors with wet ice reduced the remaining KD compared to the wet ice condition. The most rational reason for this phenomenon might be that the addition of esterase inhibitors such as PMSF could cause matrix effects and lead to ionization suppression [20]. Another plausible explanation for the decreased remaining KD in whole blood might be an altered blood-to-plasma distribution/partitioning process caused by esterase inhibitors [21,22]. Similarly, lowering pH by either FA or AA did not have a synergistic effect on stabilizing KD in dog whole blood. Considering the fact that pH significantly affects esterase activities compared with temperature [23], we proposed that the major reason for instability of KD in dog whole blood was probably due to chemical hydrolysis rather than esterase-mediated degradation. Therefore, neither adjusting pH to a certain level beyond the enzymatic working pH window nor the addition of esterase inhibitors could effectively minimize degradation of KD.

**Figure 3:**
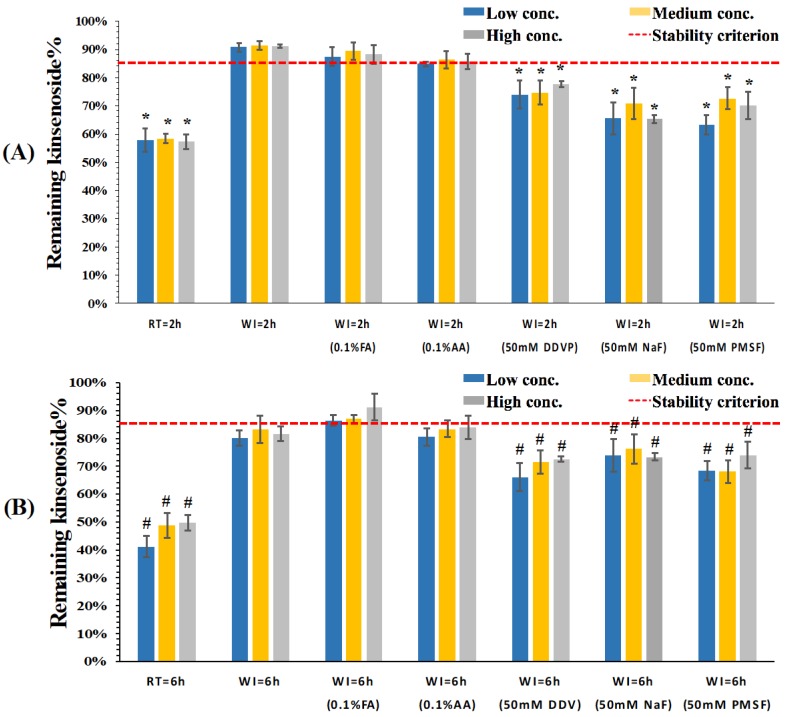
Remaining percentage of kinsenoside in beagle dog plasma after (**A**) 2 h and (**B**) 6 h incubation under room temperature and wet ice conditions (*n* = 3). The red dash lines indicate the stability criterion (85% of the nominal analyte concentrations). FA: formic acid; AA: acetic acid; DDVP: 2,2-Dichlorovinyl dimethyl phosphate; NaF: sodium fluoride; PMSF: phenylmethanesulfonyl fluoride. * *p* < 0.05 compared with plasma samples incubated on wet ice after 2 h; # *p* < 0.05 compared with plasma samples incubated on wet ice after 6 h.

The stability of KD in dog plasma was similarly investigated. Temperature was also found to play a critical role in stabilizing KD in dog plasma. As shown in Figure 3A, lowering the temperature could adequately stabilize KD in dog plasma for up to 2 h and more than 90% KD remained when the plasma samples were incubated on wet ice. Moreover, the stability of KD in neutral and acidified plasma was similar. When the incubation time was prolonged up to 6 h, as shown in Figure 3B, the percentage remaining of KD in neutral and acidified plasma samples incubated on wet ice was around 85%, suggesting that both sample treatment time and temperature should be strictly controlled.

### 3.3. Method Validation

**Figure 4:**
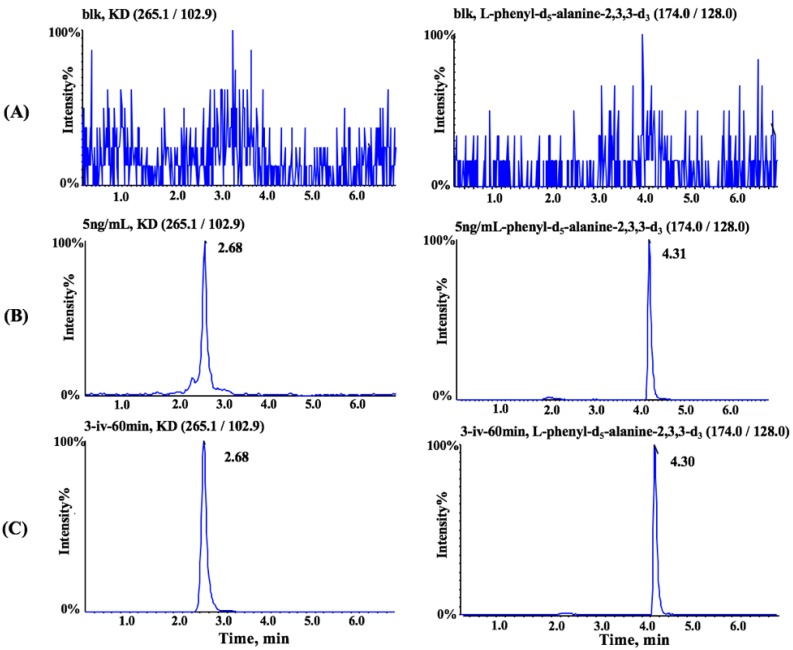
Typical multiple reaction monitoring (MRM) chromatograms of kinsenoside (**left**) and internal standard (**right**) in (**A**) blank dog plasma, (**B**) plasma sample at the LLOQ level, and (**C**) plasma sample obtained 1 h after intravenous administration of 3 mg/kg of kinsenoside.

The specificity was evaluated by comparing chromatograms of six different lots of blank dog plasma with corresponding blank samples spiked with the analyte and the IS at the LLOQ level. No interfering peaks were observed at the retention time of both analyte and IS. The typical chromatograms of extracted blank dog plasma, processed samples spiked with the analyte, and the IS at the LLOQ level as well as plasma sample obtained 1 h after intravenous administration of 3 mg/kg of KD are shown in Figure 4. The LOD and LLOQ were found to be 2 and 5 ng/mL, respectively. The calibration curves were linear over the concentration range of 5–2000 ng/mL for KD. The correlation coefficients for all calibration curves were >0.99. The deviation of the backcalculated values from the nominal standard concentrations were less than 15%.

The accuracy and precision data of the method are summarized in Table 1. The intra-day accuracy and precision were 97.43–103.67% and ≤5.02%, respectively, while the inter-day accuracy and precision were 96.78–104.07% and ≤5.52%, respectively. The results of both accuracy and precision were all within the acceptance criteria, which confirmed that this assay was reproducible and reliable for quantification of KD in dog plasma samples. A summary of extraction recoveries and matrix effects of the analyte is shown in Table 2. The recovery of KD at all QC levels ranged from 79.92% to 86.14%. Furthermore, satisfactory results of matrix effect were obtained for the analyte. The extraction recovery and matrix effect of IS were found to be 84.75% and 97.55%, respectively. Therefore, no co-eluting substance influenced the ionization of the analyte and IS. No carry-over was observed after analysis of the highest concentration of calibration standard (data not shown). The accuracy and precision of the dilution integrity indicated that dilution with blank plasma did not affect accurate determination of samples with concentration above the limit of quantification (data not shown).

The stability test results showed that the plasma samples were stable on wet ice for 2 h, at −80 °C for 21 days, and after three cycles of freezing and thawing (Table 3). There was also no significant degradation during the storage of processed samples in the autosampler for 24 h. All stock and working solutions were found to be stable in ACN–water (1:1, *v*/*v*) under their corresponding storage conditions (data not shown).

### 3.4. Preclinical Bioavailability Study

**Figure 5:**
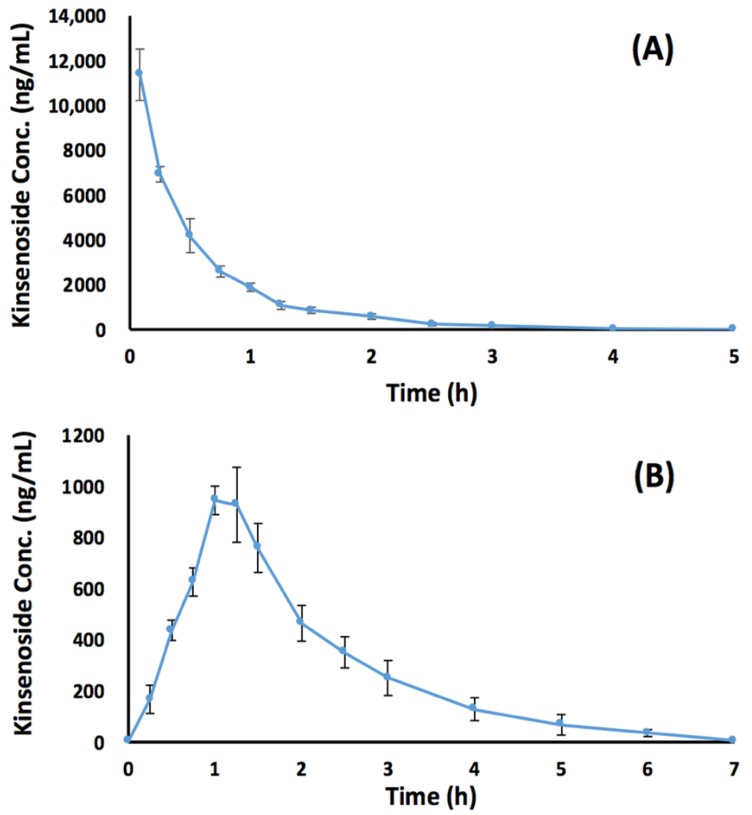
Plasma concentration versus time profiles in beagle dogs following (**A**) intravenous administration and (**B**) oral administration of 3 mg/kg kinsenoside. Data represent mean ± SD obtained from six beagle dogs.

The plasma concentration–time profiles of KD following intravenous and oral administration are presented in Figure 5. Since plasma samples collected from the intravenous group at 0.083, 0.25, 0.5, and 0.75 h were above the upper limit of quantification, such samples were diluted 10-fold with blank dog plasma prior to LC-MS/MS analysis. The major pharmacokinetic parameters are listed in Table 4. After an oral dose of 3 mg/kg, KD was quickly absorbed and the C_max_ was reached at around 1.04 h, followed by a rapid elimination with a half-life of 0.915 h. The mean AUC_0-t_ values were 6820 and 1880 ng·min/mL for intravenous and oral doses of 3 mg/kg KD. Based upon such data, the bioavailability of KD following oral administration (3 mg/kg) was found to be 27.6%. Compared with those of the majority of extensively researched isoflavone glycosides, the bioavailability of KD was at least 4-fold higher [24,25,26]. One of the plausible reasons for this is that KD has been reported to be stable against liver microsomal enzyme-associated metabolism, indicating that KD was not readily metabolized in the liver and, thus, a greater fraction of KD could reach the general circulation unchanged [10]. Another possible reason might be that KD lacks intestinal metabolism and possesses excellent absorption through the gastrointestinal tract. Although this study provides the groundwork for knowing the oral bioavailability of KD, comprehensive studies are required to further investigate absorption, distribution, metabolism, and excretion processes in preclinical animal models.

## 4. Conclusions

An improved LC-MS/MS method for the determination of KD in dog plasma was reported after elaborate investigation of analyte stability as well as intensive optimization of sample preparation and chromatographic conditions. The stability of KD in beagle dog whole blood and plasma was systematically evaluated. Using optimized sample preparation and chromatographic conditions, the bioanalytical method was validated and successfully applied to preclinical pharmacokinetic and bioavailability study of KD in beagle dogs. The bioavailability of KD after oral administration was reported for the first time. In summary, this study provides valuable pharmacokinetic information for the development of a suitable dosage form of KD.

## Figures and Tables

**Table 1:** Intra-day and inter-day accuracy and precision of kinsenoside in beagle dog plasma.

Run	Nominal Conc. (ng/mL)	Calculated Conc. (ng/mL)	Accuracy (%)	Precision (% RSD)
Intra-day (*n* = 6)	5	4.87 ± 0.19	97.43	3.82
	15	15.55 ± 0.71	103.67	4.54
	150	154.50 ± 7.76	103.00	5.02
	1500	1595.00 ± 41.93	100.78	4.67
Inter-day (*n* = 18)	5	4.84 ± 0.27	96.78	5.52
	15	15.61 ± 0.68	104.07	4.34
	150	152.61 ± 7.62	101.74	4.99
	1500	1547.78 ± 66.13	103.19	4.27

**Table 2:** The recovery and matrix effect of kinsenoside and internal standard.

Compound	Nominal Conc. (ng/mL)	Extraction Recovery (*n* = 6)		Matrix Effect (*n* = 6)	
		Mean (%)	RSD (%)	Mean (%)	RSD (%)
kinsenoside	15	79.92	3.67	93.01	5.66
	150	86.14	5.81	101.49	3.71
	1500	80.90	3.17	95.27	3.08
IS	500	84.75	4.88	97.55	4.44

**Table 3:** Summary of kinsenoside stability in beagle dog plasma under various storage conditions (*n* = 3).

Stability	Nominal Conc. (ng/mL)	Calculated Conc. (ng/mL)	Accuracy (%)
Plasma samples on wet ice for 2 h	15	14.27 ± 0.74	95.11
	150	140.80 ± 2.98	93.87
	1500	1374.13 ± 24.63	91.61
Plasma samples stored at −80 °C for 21 days	15	13.63 ± 0.36	90.89
	150	137.67 ± 2.62	91.78
	1500	1406.67 ± 26.25	93.78
Plasma samples after three freeze–thaw cycles	15	13.43 ± 0.46	89.53
	150	136.33 ± 4.50	90.89
	1500	1386.67 ± 24.94	92.44
Post-preparative samples stored at 4 °C for 24 h	15	13.42 ± 0.37	89.47
	150	137.50 ± 4.99	91.67
	1500	1333.33 ± 34.60	88.89

**Table 4:** Major pharmacokinetic parameters in beagle dogs following intravenous or oral administration of kinsenoside at a dose of 3 mg/kg.

Pharmacokinetic Parameter	Intravenous Group	Oral Group
AUC_0–t_ (ng·h/mL)	6820 ± 295	1880 ± 17.6
AUC_0–∞_ (ng·h/mL)	6830 ± 295	1910 ± 42.5
C_max_ (ng/mL)	11,400 ± 996	965 ± 59.7
T_max_ (h)	0.0833	1.04 ± 0.102
λ_z_ (1/h)	1.47 ± 0.0404	0.764 ± 0.0737
t_1/2_ (h)	0.47 ± 0.0130	0.915 ± 0.0940
CL (mL/h/kg)	440 ± 18.7	NA
Vd (mL/kg)	300 ± 17.1	NA
Bioavailability	NA	27.6%

NA, Not applicable.
